# Supplementary material for: Association between Body Fat and Elevated Blood Pressure among Children and Adolescents Aged 7–17 Years: Using Dual-Energy X-ray Absorptiometry (DEXA) and Bioelectrical Impedance Analysis (BIA) from a Cross-Sectional Study in China
Source: Int J Environ Res Public Health. 2021 Sep 2;18(17):9254. doi: 10.3390/ijerph18179254 (PMC8431661; doi:10.3390/ijerph18179254)
Supplement: Supplementary file 1 [file ijerph-18-09254-s001.zip › ijerph-1339116-supplementary.pdf]

Table S1. Association between body fat percentage (%) and incidence of blood pressure level among boys and girls.

	Overall	Boys	Girls
Dual frequency BIA machine			
SBP			
Continuous	0.429(0.359,0.499)**	0.525(0.429,0.621)**	0.507(0.402,0.612)**
Q1	Reference	Reference	Reference
Q2	1.752(0.000,3.504)	2.42(0.235,4.604)*	1.800(-1.081,4.68)
Q3	3.564(1.757,5.370)**	7.346(4.933,9.758)**	2.172(-0.702,5.047)
Q4	9.775(7.955,11.594)**	11.748(9.203,14.293)**	10.097(7.234,12.959)**
p-value for trend	<0.001	<0.001	<0.001
DBP			
Continuous	0.157(0.110,0.203)**	0.141(0.077,0.205)**	0.207(0.133,0.282)**
Q1	Reference	Reference	Reference
Q2	-0.523(-1.681,0.636)	-0.804(-2.224,0.616)	0.035(-1.999,2.068)
Q3	0.589(-0.605,1.784)	1.177(-0.391,2.745)	0.508(-1.522,2.537)
Q4	3.345(2.142,4.548)**	3.585(1.931,5.240)**	3.547(1.527,5.568)*
p-value for trend	<0.001	0.012	<0.001

$^{*}p < 0.05$; $^{**}p < 0.001$

Model was adjusted for age, gender, vegetable consumption, fruits consumption, smoking habits, alcohol drinking, sleeping duration and mid-high sports hour.

Table S2. Association between body fat percentage (%) and incidence of blood pressure level among different age.

	7-12years	13-15years	16-17years
Dual frequency BIA machine			
SBP			
Continuous	0.496(0.400,0.591)**	0.531(0.404,0.657)**	0.656(0.461,0.851)**
Q1	Reference	Reference	Reference
Q2	1.880(-0.363,4.124)	1.557(-1.718,4.832)	7.940(3.484,12.397)*
Q3	4.417(2.076,6.759)**	4.176(0.746,7.605)*	11.690(6.804,16.576)**
Q4	11.105(8.697,13.513)**	11.718(8.357,15.079)**	16.267(10.920,21.615)**
p-value for trend	<0.001	<0.001	<0.001
DBP			
Continuous	0.182(0.115,0.249)**	0.153(0.071,0.235)**	0.182(0.039,0.325)*
Q1	Reference	Reference	Reference
Q2	-0.248(-1.808,1.311)	-2.214(-4.294,-0.133)*	2.045(-1.188,5.277)
Q3	1.187(-0.440,2.815)	-1.325(-3.503,0.854)	3.429(-0.115,6.973)
Q4	3.944(2.271,5.618)**	2.776(0.641,4.911)*	3.758(-0.120,7.637)
p-value for trend	<0.001	<0.001	0.013

$^{*}p < 0.05$; $^{**}p < 0.001$

Model was adjusted for age, gender, vegetable consumption, fruits consumption, smoking habits, alcohol drinking, sleeping duration and mid-high sports hour.

Table S3. Association between body fat percentage (%) and risk of EBP, ESBP and EDBP among boys and girls.

	Overall	Boys	Girls
Dual frequency BIA machine			
EBP			
Continuous	1.055(1.040,1.070)**	1.076(1.054,1.099)**	1.070(1.047,1.094)**
Q1	Reference	Reference	Reference
Q2	1.331(0.946,1.873)	1.569(0.999,2.464)	1.379(0.756,2.518)
Q3	1.497(1.055,2.125)*	2.740(1.662,4.517)**	1.336(0.735,2.430)
Q4	3.703(2.585,5.305)**	5.897(3.39,10.257)**	4.166(2.301,7.543)**
p-value for trend	<0.001	<0.001	<0.001
ESBP			
Continuous	1.058(1.043,1.073)**	1.081(1.058,1.104)**	1.075(1.051,1.100)**
Q1	Reference	Reference	Reference
Q2	1.354(0.956,1.917)	1.671(1.055,2.648)*	1.345(0.724,2.497)
Q3	1.532(1.074,2.187)*	2.846(1.712,4.731)**	1.377(0.746,2.541)
Q4	3.754(2.612,5.394)**	6.249(3.578,10.913)**	4.206(2.297,7.699)**
p-value for trend	<0.001	<0.001	<0.001
EDBP			
Continuous	1.041(1.021,1.061)**	1.036(1.007,1.067)*	1.051(1.023,1.080)**
Q1	Reference	Reference	Reference
Q2	0.933(0.545,1.597)	0.696(0.316,1.532)	1.219(0.540,2.754)
Q3	1.300(0.775,2.183)	2.039(1.016,4.093)*	1.041(0.458,2.363)
Q4	2.308(1.418,3.758)*	2.021(0.982,4.161)	2.622(1.214,5.662)*
p-value for trend	<0.001	0.017	<0.001

*$p < 0.05$; **$p < 0.001$

Model was adjusted for age, gender, vegetable consumption, fruits consumption, smoking habits, alcohol drinking, sleeping duration and mid-high sports hour.

Table S4. Association between body fat percentage (%) and risk of EBP, ESBP and EDBP among different age.

	7-12years	13-15years	16-17years
Dual frequency BIA machine			
EBP			
Continuous	1.062(1.040,1.084)**	1.090(1.060,1.12)**	1.104(1.047,1.164)**
Q1	Reference	Reference	Reference
Q2	1.534(0.945,2.488)	1.333(0.708,2.510)	4.363(1.219,15.622)*
Q3	1.922(1.175,3.145)*	1.996(1.012,3.940)*	6.937(1.596,30.157)*
Q4	4.097(2.485,6.755)**	7.938(3.850,16.369)**	16.866(3.562,79.864)**
p-value for trend	<0.001	<0.001	<0.001
ESBP			
Continuous	1.068(1.045,1.090)**	1.093(1.062,1.124)**	1.119(1.059,1.182)**
Q1	Reference	Reference	Reference
Q2	1.590(0.959,2.635)	1.465(0.774,2.775)	4.278(1.186,15.435)*
Q3	2.082(1.250,3.470)*	2.090(1.052,4.154)*	8.184(1.779,37.640)*
Q4	4.258(2.549,7.116)**	8.226(3.975,17.023)**	23.236(4.608,117.171)**
p-value for trend	<0.001	<0.001	<0.001
EDBP			
Continuous	1.044(1.016,1.072)*	1.036(0.999,1.074)	1.049(0.990,1.113)
Q1	Reference	Reference	Reference
Q2	1.021(0.495,2.109)	0.300(0.093,0.973)*	2.293(0.569,9.243)
Q3	1.815(0.917,3.595)	0.309(0.100,0.957)*	2.226(0.492,10.067)
Q4	2.508(1.280,4.915)*	1.112(0.418,2.955)	2.816(0.532,14.915)
p-value for trend	0.002	0.058	0.106

$^{*}p < 0.05$; $^{**}p < 0.001$

Model was adjusted for age, gender, vegetable consumption, fruits consumption, smoking habits, alcohol drinking, sleeping duration and mid-high sports hour.
